# Uterine Tumors Resembling Ovarian Sex Cord Tumors: Case Report of Rare Pathological and Clinical Entity

**DOI:** 10.1155/2017/2736710

**Published:** 2017-09-11

**Authors:** Rotem Sadeh, Yakir Segev, Meirav Schmidt, Jacob Schendler, Tamar Baruch, Ofer Lavie

**Affiliations:** ^1^Division of Gynecology Oncology, Department of Obstetrics and Gynecology, Carmel Medical Center, Haifa, Israel; ^2^Department of Pathology, Carmel Medical Center, Haifa, Israel

## Abstract

Uterine tumors resembling ovarian sex cord tumors (UTROSCT) are rare uterine neoplasms. These tumors are usually benign, displaying a nodular or polypoid growth pattern; common occurrence is observed at the 4th to 6th decade of life. This entity is divided according to clinical behavior and pathological typical findings including different immunohistochemical staining. Traditionally type I tumors show a predominant endometrial stromal pattern with less than 50% ovarian sex cord component. This type has been shown to behave more aggressively with a decreased disease free survival period. Type II tumors, the classical UTROSCT, are less invasive but have the tendency to recur. We report a case of a 57-year-old patient presenting with postmenopausal bleeding. Hysteroscopic polypectomy showed the diagnosis of UTROSCT. This case presents a less morbid minimally invasive treatment plan and exemplifies that in patients where low malignant potential exists and their will is taken into consideration such management is both crucial and correct.

## 1. Introduction

Uterine tumors resembling ovarian sex cord tumor (UTROSCT) is an extremely rare type of uterine neoplasm and its clinical characteristics are not fully understood. To date, only 77 cases have been reported in the English literature [1–3]. Gomes et al. initially described the concept of sex cord differentiation in a uterine tumor and categorized them into two subtypes [3, 4]. The first type termed as endometrial stromal tumor with sex cord-like elements (ESTSCLE) largely resembles traditional endometrial stromal tumors. The second type is comprised of tumors entirely composed of elements resembling sex cord tumors of ovary and is named as UTROSCT [5].

This classification is critical due to the fact that the two types of tumors resemble each other histologically and yet differ significantly in terms of clinical behavior and genetic features, thus requiring different clinical approaches [6].

Since the initial report in 1976, a distinct separation between the two subtypes has been formed. Until recent years, this categorization was solemnly clinically based [1, 2].

Recently the classification has been reframed based on several immunohistochemical markers, including Calretinin, CD99, Inhibin, and Melan [7].

Type I ESTCLE shows a predominant endometrial stromal pattern, with usually less than 50% ovarian sex cord pattern. Such tumors typically express Calretinin immunohistochemical marker. This tumor also contains unique genetic features and a characteristic translocation. These findings are absent in Type II tumor [6].

Type I is known to have a significantly higher malignant potential and the outcome is contingent upon grade and stage of the underlying stromal neoplasm [1].

Type II also known as classical URTOSCT is considered to be of low malignant potential secondary to occasional recurrence, although they typically exhibit benign biological behavior. A positive Calretinin, as well as another positive staining by Inhibin, CD 99 or Melan A, is to be obtained in order to reach the diagnosis of this specific type [5, 8]. UTROSCT usually occurs in middle-aged women. Most patients present with abnormal uterine bleeding and/or abdominal pain, along with an enlarged uterus or a palpable uterine mass [4]. Hysterectomy with or without bilateral salpingo-oophorectomy is the acceptable treatment for UTROSCT [6].

## 2. Case

A 57-year-old Caucasian woman presented with postmenopausal bleeding (PMB) and hot flushes was admitted to our department. Initial workup included physical examination and transvaginal sonography with no pathological findings.

**Figure 1:**
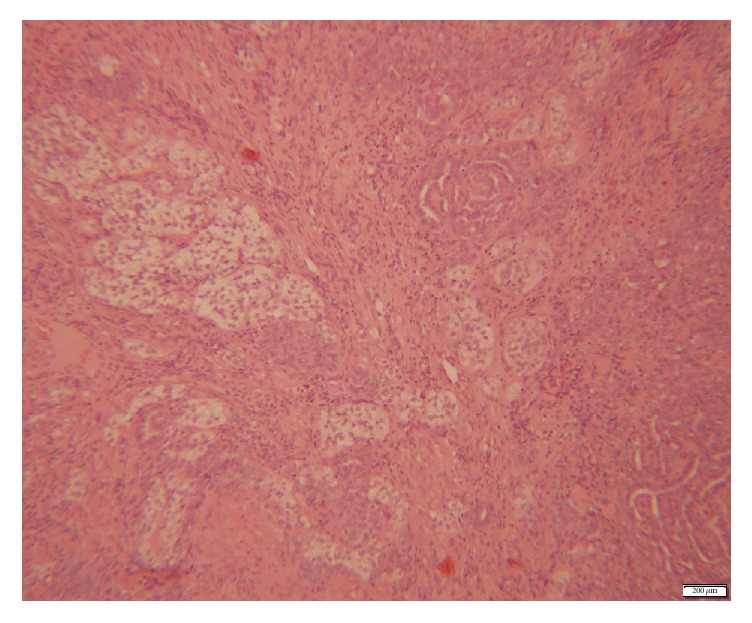
H&E microscopic image of the polyp which was composed of elements resembling ovarian sex cord tumors, including solid areas, glomeruloid structures, and small nests.

Since the presenting symptom was PMB, an endometrial aspiration (pipelle) was performed and pathologic examination revealed syncytial metaplasia. Consequently, the patient experienced a few more episodes of bleeding; therefore hysteroscopy was performed revealing normal appearing endometrium with an endometrial polyp. Eventually, polypectomy was performed. On gross examination there was an elastic pale grey mass measuring 9*∗*8*∗*2.5 mm. On microscopic examination, fragments of uterine wall infiltrated by malignant nonpleomorphic tumor cells with pale chromatin small nucleoli minor variations in their round to oval nuclear contour and rare mitosis. The cytoplasm of tumor cells is mostly scant but some epithelioid cells with abundant foamy cytoplasm are seen. These neoplastic cells form different patterns of growth: ovarian sex cord tumors, including solid areas, glomeruloid structures, and small nests (Figure 1).

**Figure 2:**
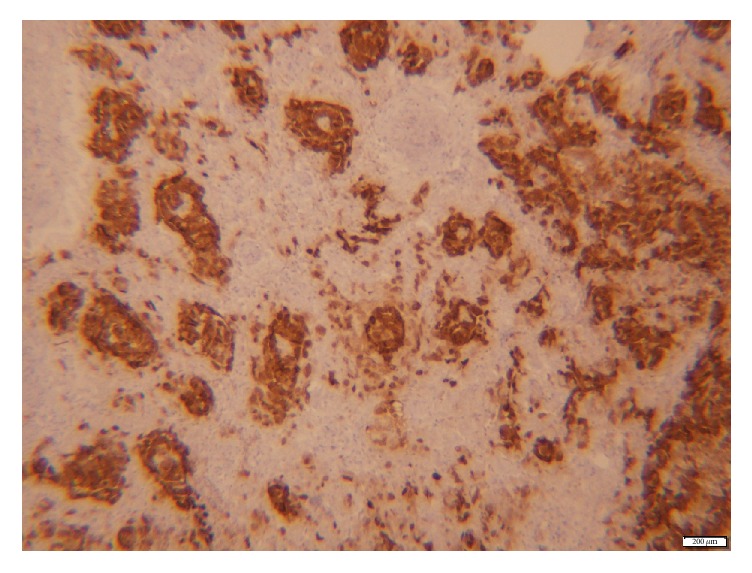
Immunohistochemistry of the tumor cells which were positive for Calretinin.

On immunohistochemistry tumor cells were positive for Calretinin (Figure 2), MART-1, Inhibin, CD 99, Desmin, Actin, Vimentin, and Pankeratin. Staining for EMA, Chromogranin, Synaptophysin, S100, CD30, CD31, CD34, CD117, Myf-4, A-FP, oct 3/4, and PLAP was all negative. Histopathological findings were consistent with uterine tumor resembling sex cord tumor.

Due to the rare histological result of the polypectomy a computerized tomography (CT) was done and showed no abnormality. No other imaging studies were performed.

Pathological results correlated with a neoplasm of low malignant potential (subtype 1). Taking into account the patient's age and personal preference she underwent laparoscopic total abdominal hysterectomy and bilateral salpingo-oophorectomy without the additional omentectomy and regional lymph node dissection.

No further macroscopic abnormal finding was observed in the operative field. Upon final pathological review, the uterus showed an inactive endometrium with focal ulceration, fibrin, and giant cell reaction consistent with previous polypectomy. No residual tumor was documented. An unremarkable cervix and lower uterine segments were observed; furthermore bilateral ovaries with atrophic changes and inclusion cysts as well as bilateral benign fallopian tube were described. Today, three years after the initial treatment the patient is being followed up with no evidence of recurrence.

## 3. Discussion

We present here an additional case report of UTROSCT. It is of utmost importance to bring forward every such patient in order to reveal the unknown complexity of this intriguing entity.

UTROSCT are rare uterine neoplasms. The differential diagnosis includes leiomyoma, endometrial polyp, and malignant neoplasm of the uterus (endometrial carcinoma and sarcoma). The initial morphologic description in the literature failed to describe whether UTROSCT represents a variant within the spectrum of endometrial stromal tumors (ESTs), which may rarely exhibit areas of sex cord-like differentiation or whether it is a distinct uterine neoplasm unrelated to ESTs [7]. The neoplasm usually occurs in middle-aged women. Most patients present with abnormal uterine bleeding and/or abdominal pain, along with an enlarged uterus or a palpable uterine mass [3]. It is now understood relatively clear that two main subtypes exist. Type I tumors which show a predominant endometrial stromal pattern with less than 50% focal ovarian sex cord component. This subtype has been tagged as ESTSCLE. These tumors also typically present cytogenetic findings which are unique to stromal tumors and are not found in the classical URTOSCT. In ESTSCLE fusion of two novel genes (JAZF1 and JJAZ1) occurs, which is not observed in UTROSCT, emphasizing that UTROSCT is another entity than ESTSCLE; however, the origin of UTROSCT remains uncertain [9]. Furthermore, it has been shown that this type has a more aggressive behavior and statistically a decreased disease free survival. Type II tumors also known as classical UTROSCT have well known histological criteria mentioned previously [5, 6]. These neoplasms present a mild clinical course and thus require a more conservative and less invasive treatment plan; nevertheless it is not unheard-of to observe recurrence even in this subtype [10]. There are no specific pathognomonic imaging findings, and the diagnosis is made exclusively on histopathologic examination. An array of architectural patterns has been introduced; these include plexiform cords, watered-silk, microfollicle, and diffuse patterns of growth [10, 11]. These findings suggest that UTROSCT may result from divergent differentiation in endometrial stromal tumors or represent a distinct group of uterine tumors with sex cord-like differentiation that are closer in histogenesis to ovarian sex cord stromal tumors [11, 12].

The most significant information from recently conducted studies concerns the immunophenotype of these lesions, especially of UTROSCT. Out of the plethora of the immunohistochemical stains, a panel of 4 stains, including Calretinin, Inhibin, CD99, and Melan A, seemed to be the most characteristic sex cord markers. Positivity for Calretinin and at least for one more of the other above-mentioned markers confirms the diagnosis of UTROSCT. Endometrial stromal tumors with sex cord-like elements, on the other hand, usually express only one of these sex cord markers, mostly Calretinin [5]. In our case report immunohistochemistry tumor cells were positive for 3 out of these four stains: Calretinin, Inhibin, and CD 99, which confirms the diagnosis. It is thus highly important to request these staining procedures in order to construct a modified custom maid treatment plan for each individual and his unique pathological findings.

Hysterectomy with or without bilateral salpingo-oophorectomy is usually the treatment for UTROSCT [2]. UTROSCT are associated with less aggressive behavior compared with ESTSCLEs, and given this fact there are no specific guidelines for the radicality of the surgical procedure, nor the need for adjuvant therapy [13].

The decision to remove the adnexa is based on the clinical status, tumor histological type, and the age of the patient. Our patient was 57 years old, with postmenopausal bleeding, and therefore, bilateral salpingectomy was performed. However, owing to the uncertain malignant potential of UTROSCT, while fertility sparing surgery in young patients could seem safe, the risks of recurrence are precipitating the need for close follow-up.

To date there are no recommendations for adjuvant chemotherapy or radiotherapy [13]. Although the prognosis of these tumors is usually favorable, we should keep in mind that these tumors may cause metastatic lesions due to malignant transformation [14]. Our patient, today, three years after treatment, is free of disease. Owing the late recurrences described in the literature, follow-up is recommended.

## 4. Conclusions

In this case report, a description of a patient with classical subtype UTROSCT was presented. We believe that the distinction between the two subtypes is fundamental for both gynecooncologists and the gynecopathologists in order to prevent overtreatment and supply adequate care for those rare patients.

This subclassification can be performed readily by requesting immunostaining in all subsequent pathological results. By doing so, treatment is proportionate and tailored on an individual basis. We also urge that the importance of bringing forward such similar cases in the future thus strengthens our clinical practice and management.
